# Multimodality Imaging of the Anatomy of the Aortic Root

**DOI:** 10.3390/jcdd8050051

**Published:** 2021-05-04

**Authors:** Vera Lucia Paiocchi, Francesco F. Faletra, Enrico Ferrari, Susanne Anna Schlossbauer, Laura Anna Leo, Francesco Maisano

**Affiliations:** 1 Division of Cardiology, Fondazione Cardiocentro Ticino, Via Tesserete 48, CH-6900 Lugano, Switzerland; vera.paiocchi@cardiocentro.org (V.L.P.); enrico.ferrari@cardiocentro.org (E.F.); susanne.schlossbauer@cardiocentro.org (S.A.S.); lauraanna.leo@cardiocentro.org (L.A.L.); 2 Cardiac Surgery, Università Vita Salute San Raffaele, via Olgettina 58, 20132 Milano, Italy; francesco.maisanopersonale@gmail.com

**Keywords:** aortic root, echocardiography, computed tomography (CT), cardiac magnetic resonance (CMR)

## Abstract

The aortic root has long been considered an inert unidirectional conduit between the left ventricle and the ascending aorta. In the classical definition, the aortic valve leaflets (similar to what is perceived for the atrioventricular valves) have also been considered inactive structures, and their motion was thought to be entirely passive—just driven by the fluctuations of ventricular–aortic gradients. It was not until the advent of aortic valve–sparing surgery and of transcatheter aortic valve implantation that the interest on the anatomy of the aortic root again took momentum. These new procedures require a systematic and thorough analysis of the fine anatomical details of the components of the so-called aortic valve apparatus. Although holding and dissecting cadaveric heart specimens remains an excellent method to appreciate the complex “three-dimensional” nature of the aortic root, nowadays, echocardiography, computed tomography, and cardiac magnetic resonance provide excellent images of cardiac anatomy both in two- and three-dimensional format. Indeed, modern imaging techniques depict the aortic root as it is properly situated within the thorax in an attitudinally correct cardiac orientation, showing a sort of “dynamic anatomy”, which admirably joins structure and function. Finally, they are extensively used before, during, and after percutaneous structural heart disease interventions. This review focuses on the anatomy of the aortic root as revealed by non-invasive imaging techniques.

## 1. Introduction

Cardiac anatomy is usually taught to medical students on the basis of examination of the cadaveric heart specimens. Indeed, holding and dissecting a human heart remains an excellent method to appreciate the complex “three-dimensional” anatomy of the heart. However, it must be recognized that cardiac anatomy is, “per se”, extremely complex and not always does it become part of the student’s cultural background, especially when it is learned in the first years of medical school as a small part of the body’s anatomy. In addition, anatomical exploration is done in the flaccid, cadaveric heart. Today, echocardiography, computed tomography (CT), and cardiac magnetic resonance (CMR) provide excellent images of cardiac anatomy both in two- and three-dimensional format. Although these techniques are mainly used to discover pathological features, they also offer exceptional images of normal cardiac anatomy. For many aspects, these techniques can be considered nowadays as a kind of novel “anatomical gold standard” for the following reasons: First, differently from explanted hearts, these techniques depict the cardiac anatomy as it is properly situated within the thorax in an attitudinally correct cardiac orientation. Second, they have the unique ability to show “live” images from the beating heart, a sort of “dynamic anatomy”, which admirably joins structure and function. Third, the rapid progression of percutaneous structural heart disease interventions highlights the need to revisit cardiac anatomy using a multimodality approach. These non-invasive techniques have reached an optimal agreement with anatomy. Indeed, their accuracy in providing fine anatomic details has been verified in several studies by comparing side-by-side anatomic specimens and non-invasive images [1,2,3,4,5,6,7,8,9]. This review focuses on the anatomy of the aortic root as revealed by non-invasive imaging techniques.

## 2. Aortic Root

The fine details of the “aortic root complex” have been often overlooked by the cardiology community. Two main reasons explain this sort of “disinterestedness” towards this complex structure: (a) for a long time, the aortic root has been considered a simple and inert unidirectional conduit between the left ventricle (LV) and the ascending aorta; (b) because aortic leaflets do not have an extensive attachment with the ventricular myocardium, their motion was thought to be entirely passive—just driven by fluctuations of LV–AO gradients. The advent of aortic valve–sparing surgery and transcatheter aortic valve implantation (TAVI) techniques renewed the interest on the anatomy of the aortic root, since both procedures require a systematic and thorough analysis of the fine anatomical details of the components of this valve apparatus [10,11].

## 3. Terminology

There is a remarkable variability in the terms used to describe the aortic root components. Table 1 clarifies the terminology for everyday use.

### 3.1. The Function of the Aortic Root

The aortic root is a highly sophisticated and complex apparatus, which has been forged by millions of years of evolution to function properly for a lifetime in the most challenging physical environment. The functions of the aortic root are described in Table 2. Preserving leaflet integrity is probably the most relevant function of the aortic root. It must be said that leaflets are living structures subjected to a natural biological turnover. Their structural micro-architecture is continuously renovated, and the micro-injuries are self-repaired. Nevertheless, leaflet integrity and durability are highly dependent on the stress applied to the tissue during the closure and opening phases. The aortic root provides an extraordinary “low-stress environment” that favors leaflet durability [14,15,16].

### 3.2. Aortic Root and Surrounding Structures

**Figure 1:**
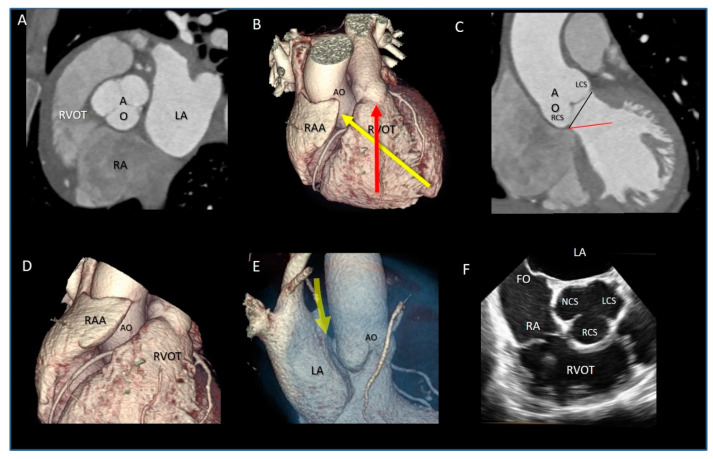
(**A**) Computed tomographic image in multiplanar modality, showing the aortic root (AO) is the cardiac centerpiece, surrounded by the atria and the right ventricular outflow tract (RVOT). (**B**) The 3D volume rendering CT image in antero-posterior projection showing the obliquity of the aortic root (yellow arrow), sited posterior and rightward to the RVOT (red arrow). (**C**) Computed tomographic in multiplanar imaging modality showing the correct attitudinal orientation of the aorta. The black line, that joins the nadir of aortic sinuses, is near 30° tilted in respect to the horizontal red line, so that the left coronary sinus (LCS) and its leaflet are at the highest position among the right coronary (RCS) and non-coronary sinuses. (**D**) CT image in 3D volume rendering modality in antero-lateral projection showing the AO is surrounded laterally by the right atrial appendage (RAA). (**E**) CT image in 3D volume rendering modality in lateral projection, showing the space between the left atrium (LA) and the AO (yellow arrow) named “sinus transversum” filled up by epicardial adipose tissue. (**F**) The 2D transesophageal echocardiography in short-axis aortic view, showing the close proximity between the fossa ovalis (FO) and the non-coronary sinus (NCS) (see text).

The AO is the cardiac “centerpiece” partially wedged between the atrioventricular orifices. (Figure 1A). Viewed from an antero-posterior plane, the aortic root is positioned to the right and posterior in respect to the right ventricular outflow tract (RVOT). The angle between the aortic root and the RVOT is approximately 40–60° (Figure 1B), while the line joining the nadir of the aortic sinuses lies in a plane that is tilted on its left of 30° in respect to the horizontal line. As a consequence, the left coronary sinus and its leaflet are at the highest position among the three sinuses (Figure 1C). On its right lateral aspect, the aortic root is surrounded by the right atrial appendage (Figure 1D), while posteriorly a space, called “sinus transversum”, separates the AO from the left atrial cavities. Adipose tissue is located at both the aortic and atrial side of the sinus (Figure 1E). The close proximity of the non-coronary sinus to the fossa ovalis (FO) is well known to interventionalists. Indeed, puncturing the FO or implanting a device for a patent foramen ovalis or secundum atrial septal defect closure may injure the aortic wall, especially if the aortic root is enlarged [17] (Figure 1F). In conclusion, the AO comes into contact with the right atrium, left atrium, interatrial septum, RVOT, mitral valve (aorto–mitral continuity), pulmonary valve, tricuspid valve, and conduction system (see below).

### 3.3. The Components of the Aortic Root

The aortic root is composed of five key components: the ventricular–arterial junction (VAJ), the crown-shaped hinge of the semilunar leaflets (also called annulus), the leaflets, the sinuses, and the sinutubular junction (STJ).

#### 3.3.1. The Ventricular–Arterial Junction (VAJ)

The VAJ refers to an area where the left ventricular outflow tract (LVOT) joins the fibro-elastic wall of the AO. Differently from the right VAJ, which is entirely muscular, the left VAJ is in part muscular and in part fibrous.

#### 3.3.2. The Muscular Component of VAJ

**Figure 2:**
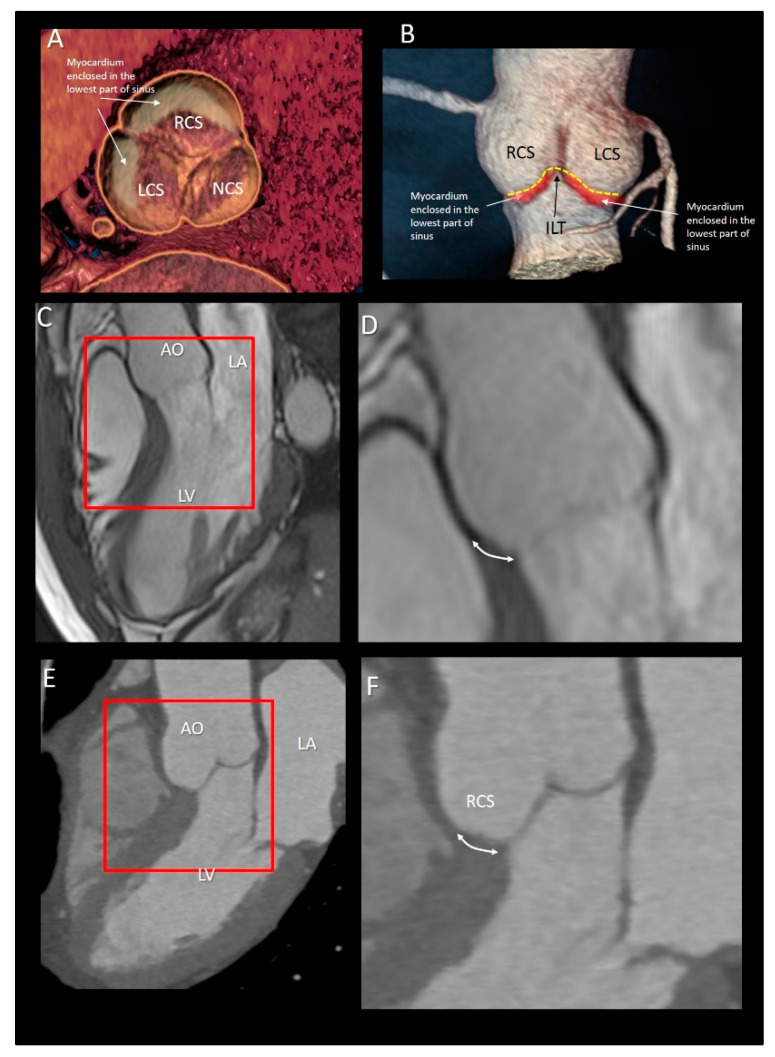
(**A**) CT image of short-axis view of the aortic root from an aortic perspective. The gray-beige areas superimposed to the image indicate the myocardium enclosed at the bases of right coronary sinus (RCS) and left coronary sinus (LCS). (**B**) CT scan of volumetric image of the aortic root. The ventriculo–arterial junction is indicated by the yellow dotted line. The red areas below indicate the muscular myocardium enclosed in the bottom part of the sinuses. (**C**) Cardiac magnetic resonance showing the left ventricle (LV), the left atrium (LA), and the aorta (AO) in cross-section long-axis view. (**D**) Magnified image of the structures in the red square of the panel C. The double-headed arrow marks the extension of muscular sleeve on the right coronary sinus (RCS). (**E**) Computed tomography showing the LV, the LA, and the AO in cross section long-axis view. (**F**) Magnified image of the structures in the red square of the panel C. The double-headed arrow marks the extension of muscular sleeve on the RCS.

The muscular tissue of VAJ represents nearly 45% of the entire VAJ. Interestingly, there is not an abrupt transition line between the ventricular myocardium and the aortic sinuses, on the contrary, the muscular component of the LVOT irregularly extends a few millimeters above the hinge lines of the right coronary leaflet (RCL) and the anterior part of left coronary leaflet (LCL) [18]. The hinge lines of the posterior part of the LCL and the non-coronary leaflet (NCL) are lacking myocardial fibers being in continuity with the anterior mitral leaflet and membranous septum. Hasdemir et al. [18] studied 95 human hearts, analyzing longitudinal strips of tissue containing each cusp, aortic, and pulmonary artery walls and left and right ventricular outflow tract. They found muscular fibers in 7 out of 95 aortic strips (7%) in continuity with the underlying LVOT muscle. In around 70% of cases, myocytes were mixed with fibrosis and fatty tissue. The arrangement where electrically active muscular fibers cover the distal part of the vessels in their junction with the muscular myocardium can also be found in the pulmonary veins [19], where they may trigger abnormal electrical activity and atrial fibrillation. Since a subgroup of outflow tract ventricular tachycardias originate from the aortic VAJ, it can be speculated that, similarly to the muscular sleeves around the pulmonary veins, the irregular mixture of electrically active myocytes, fibrosis, and fat may trigger ventricular arrhythmias [20,21,22,23]. In case ventricular arrhythmias require radiofrequency catheter ablation, an in-depth understanding of the anatomic complexity of this region may help maximize ablation success. At the same time, the electrophysiologist should be aware of potential complications when operating in this area (Figure 2).

During the ventricular filling phase and the isovolumetric contraction, the muscular component of the VAJ expands (together with the STJ), making the aortic root larger in order to accommodate the stroke volume. This expansion reduces the area of leaflet coaptation and at the time of their opening, the leaflets are in apposition only with their free edge, reducing the leaflet-to-leaflet friction. During the ejection, the VAJ contracts, while the STJ continues to expand. The shape of the aortic root becomes a truncated cone with the smaller opening toward the ventricle facilitating the progression of the blood column towards the ascending aorta. The fine sequential movement of the root components during systolic ejection resembles the peristalsis in the gut, and it is meant to optimize the efficiency of the system and reduce the stress on the structures [24].

### 3.4. The Fibrous Component of VAJ

The fibrous component represents nearly 55% of the entire VAJ and includes the mitral–aortic (MA) curtain, a fibrous avascular area located between the left (LCS) and the non-coronary sinus (NCS), and the membranous septum (MS) located between the NCS and the right coronary (RCS) sinuses.

**Figure 3:**
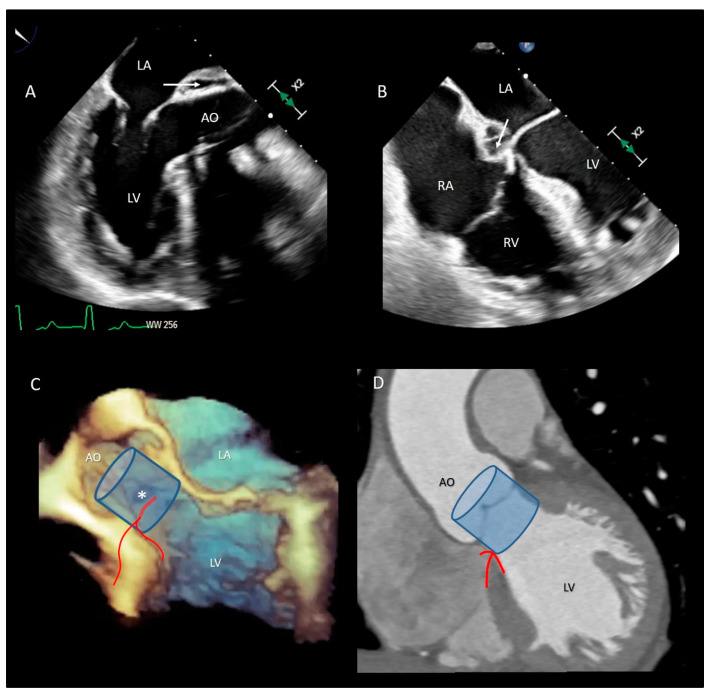
(**A**) The 2D TEE in long axis-view showing a peri-annular aortic abscess (arrow). (**B**) Same case of panel A showing the abscess may extend posteriorly on the crux cordis (arrow). (**C**) The 3D TEE in long-axis view. The red curve lines mark the position of His bundle immediately under the MS (asterisk) and its bifurcation. (**D**) CT multiplanar reconstruction showing the course of the His bundle and its branches (red lines). The light blue cylinders are representative of the position of the implanted valve. AO = aorta; LV = left ventricle; LA = left atrium; RA = right atrium; RV = right ventricle.

The MA curtain is well known to surgeons and cardiac imagers. Indeed, this area is often a target of aortic endocarditis (either on native leaflets or on aortic prostheses). The infection propagates by direct continuity of the aortic infected tissue or by infected regurgitant jet striking this area. The uncontrolled infection usually perforates the MA curtain, dissecting the aortic wall and propagating around the aortic root. A direct communication with the LVOT is often present, while a true enclosed abscess cavity very rarely develops and often a fistulous communication to other cardiac chambers can be recognized. In almost all cases the diagnosis is made by 2D TEE [25]. The peri-annular aortic abscess is a life-threatening condition since it may disrupt the fibrous skeleton and the atrioventricular septum, making the reconstructive procedure very complicated (Figure 3A,B) [26,27,28].

**Figure 4:**
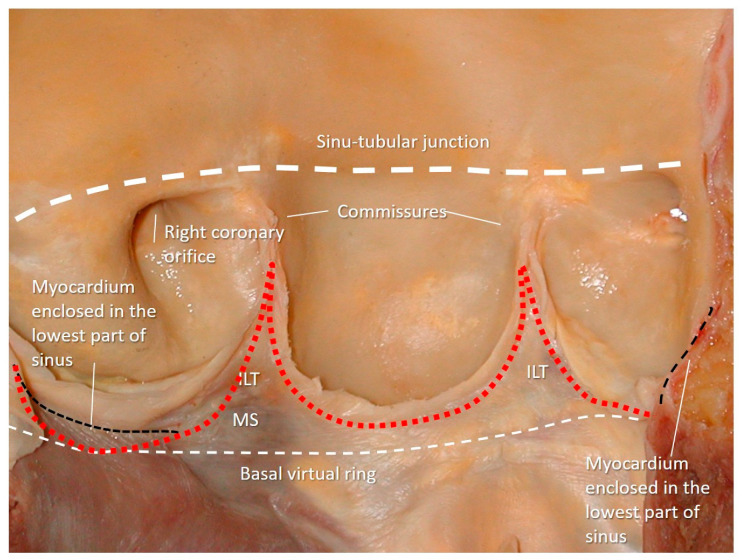
Anatomic specimen components of the aortic root: the sinus–tubular junction (white thick dotted line), the crown-shaped annulus (red dotted line), and the ventricular–arterial junction (black dotted line). The virtual basal ring (white thin dotted line). MS = membranous septum; ILT = interleaflet triangle.

Transcatheter implantation of the aortic valve (TAVI) is now recognized as state-of-the-art for patients with severe aortic stenosis and high risk for surgery. Recently, increased operator experience and enhanced transcatheter valve systems have led to a worldwide trend to use TAVI in patients who are at intermediate or low risk [29]. However, TAVI has an increased risk of injuring the atrio-ventricular conduction system. This is due to the close anatomical relationship between the aortic valve and the heart conduction system. Three major variants of AV nodes have been described, with 50% of individuals exhibiting a relatively right-sided AV bundle and 30% with a left-sided AV bundle, whereas in about 20% of patients the bundle courses under the membranous septum just below the endocardium. The last two above-described variants may expose patients to a higher risk of TAVI-induced conduction disturbances, especially in patients with a short membranous septum [29]. Today, the imaging of the membranous septum by CT scan prior to TAVI is a standard step to assess the risk of AV block after TAVI. The longer the length of the membranous septum, the lower the risk of post-TAVI AV conduction abnormalities [30]. According to the valve academic consortium, the development of post-procedural conduction disturbances may not be the strongest predictor of mortality but has a significant influence on long-term prognosis and the patient’s quality of life [31] (Figure 3C,D). Figure 4 shows an anatomic specimen with the components of the VAJ.

### 3.5. The Aortic Annulus

**Figure 5:**
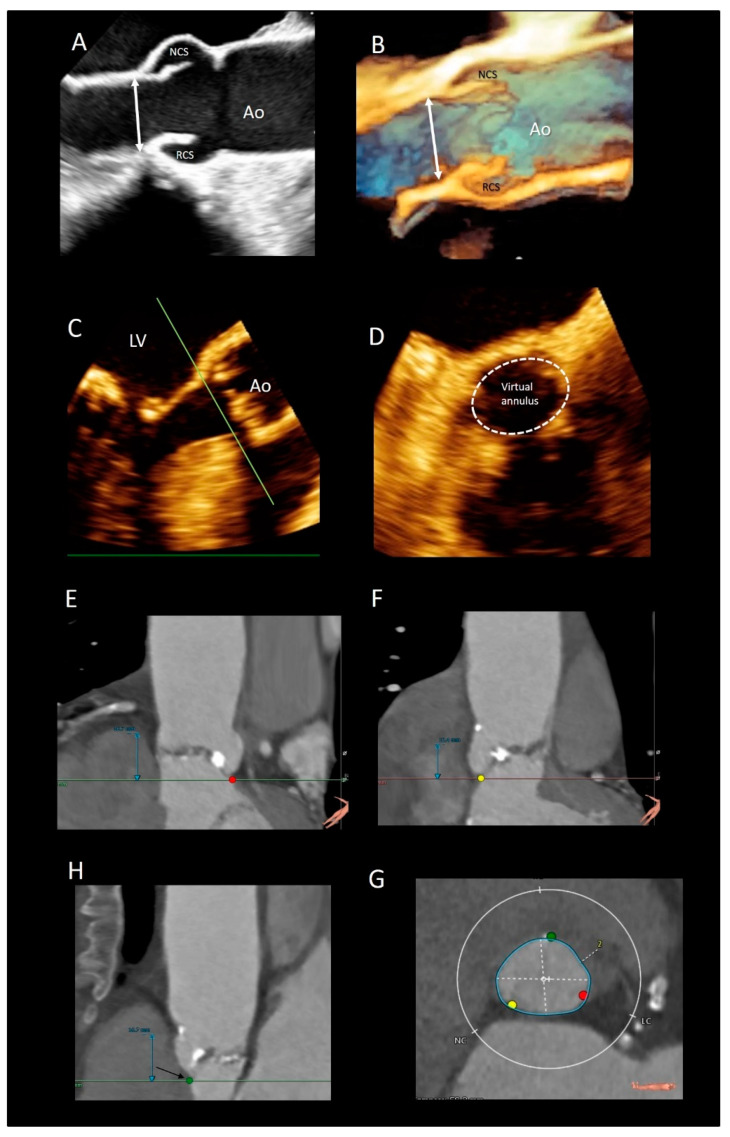
(**A**) The 2D and (**B**) 3D TEE long-axis view showing the echocardiographic annulus. (**C**,**D**) Multiplanar reconstruction using the 3D data set showing the virtual annulus. (**E**,**F**,**H**) CT scans showing the 3 nadirs (marked by the red, green, and yellow circles). (**G**) Dedicated software aligns the three nadirs in the same plane making possible measurements of diameters (white dotted lines) and circumference (blue line).

There are different interpretations of the aortic annulus depending on whether you are an echocardiographer, a cardiac surgeon, or a pathologist. Since the beginning of the bidimensional echocardiographic era, annular measurements have been routinely performed on 2D TEE in the parasternal long-axis view. More recently, using 2D TEE, the aortic annuls is measured in a 120–140° long-axis view of the ventricle. These measurements are taken on mid systole at the basal attachment of adjacent leaflets. In the TAVI era, a “virtual” annulus is obtained by joining the lower points of the three leaflets (the term “virtual” refers to the fact that this “circumferential” annulus does not exist neither anatomically nor histologically). When measured with CT multiplanar imaging or with 3D TEE, this “virtual annulus” has an elliptic shape in diastole and a more circular shape in systole. Interestingly, since the lowest points of the three leaflets do not lie on the same plane, dedicated software reconstructs the virtual annuls as if it was lying in one plane. Though CT is considered the main imaging modality for identifying the correct size of the prosthesis [32], 3D TTE may be a valid imaging alternative in patients unsuitable for MDCT. Tamborini et al. [33] showed that 3D TTE measurements were highly reproducible, and the agreement between 3D TTE and CT was very good (k = 0.84, *p* < 0.001) (Figure 5).

Even the so-called “surgical anulus” does not exist as a specific anatomical entity, once the diseased leaflets are removed, the prosthetic valve is sutured in a sort of “ring area”, which lies between the lowest part of the native leaflet hinge line and midway to the commissures. However, referring to this ring area as “annulus” is not illogical since it provides a precise reference point when the prosthesis is sutured in a supra-annular position [34].

**Figure 6:**
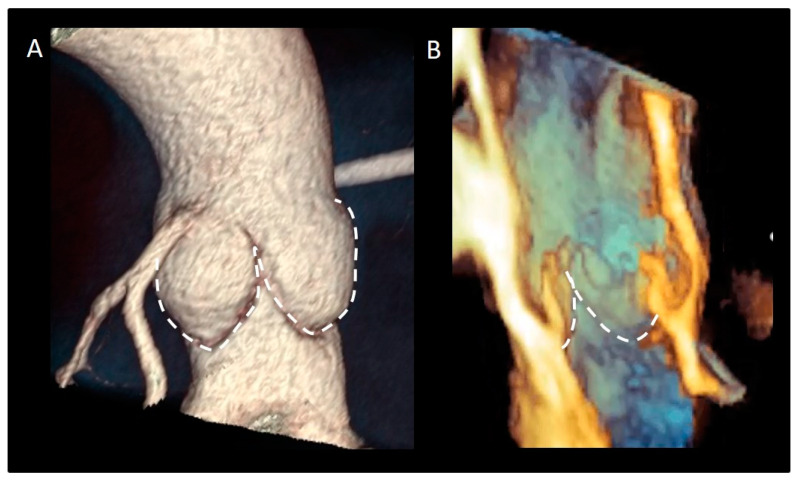
(**A**) CT in volume rendering format and (**B**) 3D TEE showing the typical crown-shaped configuration of the anatomical annulus (dotted line).

Finally, from an anatomical point of view, the aortic annulus is a geometrically complex three-dimensional crown-shaped structure. The insertion of the leaflets on the aortic wall, in fact, takes the form of three prolonged coronets with the lowest part (called nadir) lying slightly below the ventricular–arterial junction and the highest point joining the sinutubular junction. The highest joining point of two adjacent leaflets is named commissure. This anatomical arrangement guarantees a great flexibility that allows the anulus to expand during the systole minimizing the transvalvular gradient [35]. This flexibility would not be possible in the presence of a circular fibrous ring, which should be robust and rigid enough to withstand the diastolic aortic pressure. Such a ring-shaped annulus, on the other hand, would have probably resulted in a fixed transvalvular gradient comparable to that present across a mechanical or a biological prosthesis (Figure 6).

### 3.6. The Interleaflets Triangles

**Figure 7:**
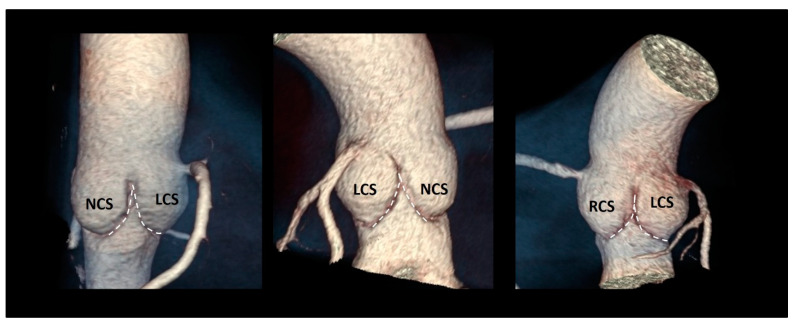
The three inter-leaflets triangles visualized with CT volume rendering format (dotted line). NCS = non-coronary sinus; LCS = left coronary sinus; RCS = right coronary sinus.

The ventricular aspect of the aortic root is lined by three fibrous triangular extensions, filling the space between the hinge line of leaflets and extending from the VAJ to the commissures [36]. The triangle between the left and right coronary leaflets is usually the smallest, while the triangle between the left and the non-coronary leaflets is in continuity with the mitral–aortic curtain. The surgical relevance of this latter triangle relies on the fact that, in a small aortic annulus (i.e., an annulus that cannot house a prosthesis size ≥21 mm), the annulus enlargement to accommodate a larger valve prosthesis may involve this anatomical portion of the root [37]. The surgical procedure consists in extending the aortotomic incision posteriorly through the commissure at the left and non-coronary sinus, cutting this triangle up to the mitral–aortic curtain. Finally, the triangle between the non-coronary and the right coronary leaflets includes the MS, which marks the site of the atrioventricular conduction bundle. Interestingly, all inter-leaflet triangles, despite anatomically considered part of the aortic root, are subjected to ventricular pressures because they remain below the aortic valve leaflets (Figure 7).

### 3.7. The Sinuses of Valsalva

**Figure 8:**
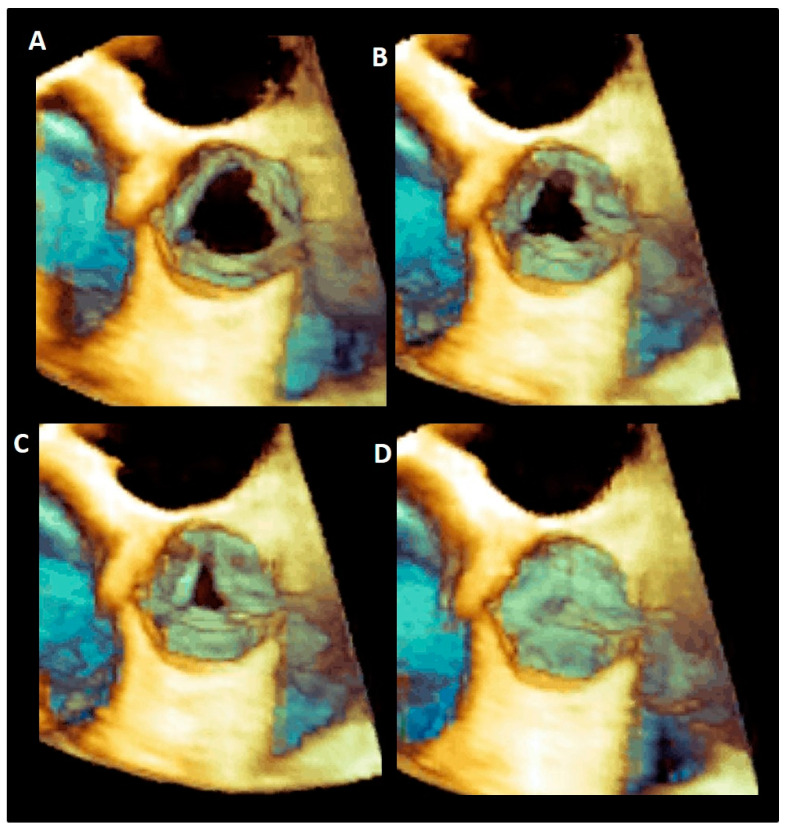
(**A**–**D**) Tridimensional 3D TEE showing the progressive closure of aortic leaflets during the systole.

The sinuses of Valsalva are bulges of the aortic root, one for each cusp in the tricuspid aortic valves. They are named according to the coronary arteries arising from them as right (RCS), left (LCS), and non-coronary (NCS) sinuses, and have different sizes, with the NCS being the largest and the LCS the smallest. One function of the aortic sinuses is to provide enough space behind the open leaflets to prevent the occlusion of the coronary artery orifices. However, the main function of the sinuses is to preserve the integrity of the aortic leaflets [38]. The most peripheral layers of blood flow, sliding along the ventricular surface of the leaflets during systole, encounter the “bottle neck” of the sinutubular junction. These peripheral layers are then forced back into the space between the open leaflets and the sinuses. By entering this space, the back flow creates a vortex that promotes a slow closure motion of leaflets, which approximate each other during the systole. At the end of the systole when the forward systolic flow still crosses the valve, the free margins of the leaflets are already very close to each other. At the beginning of the diastole, when the blood flow reverses, the distance between the valve leaflets is minimal, ensuring a synchronous, homogeneous, and stress-free leaflet closure [39] (Figure 8).

Moreover, the presence of Valsalva sinuses may guarantee a progressive increase in the valve area and therefore an absence of gradient every time the cardiac output is increased (i.e., during an intense physical activity) [40]. During the systole, in fact, a progressive reduction of the pressure inside the sinuses (induced by the vortices) favors the increased flow across the valve. In other words, the lower pressure beyond the leaflets explains how the leaflet can reach and keep a wider opening area in an increased cardiac output maintaining a relatively low gradient. In the absence of sinuses, the aortic valve appears to be progressively more obstructive to the blood flow when the output increases. Indeed, the leaflets have less space for moving to their full opening position, and their free margin will show wrinkles and folds that might increase gradients and potentially contribute to an accelerated leaflet’s degeneration [41,42,43].

The elastic capability of the sinuses to distend under pressure is another relevant characteristic that preserves the leaflets integrity. A cross-section in the middle of the body of the sinuses reveals that, in diastole, the sinuses move outward assuming a three-lobed arrangement. This diastolic configuration splits the aortic root in three almost circular sinus–leaflet assembly units, which are formed by the sinus and the corresponding leaflet and divided by the inter-leaflets’ triangles. The stress on the leaflets in diastole is almost four times than in the sinuses, and this would tether the sinuses’ wall inwards. If this does not happen, it is because these sinus–leaflet assembly units adapt, at every single beat, to the diastolic stress conditions on leaflets by reducing the “radius of curvature” of any single sinus (in other words the sinus–leaflet assembly becomes approximately spherical). This allows the share of the diastolic circumferential stress between the leaflet and the sinus becoming an enclosed self-sustained unit that contains the pressure within. This stress sharing preserves the longevity of the aortic leaflets [39,41,42]. Interestingly, in case of a bicuspid aortic valve, this sophisticated stress-sharing architecture, resulting from millions of years of evolution, is lost. It is unsurprising that these valves are predisposed to an additional circumferential stress at an early calcification and a worse outcome [43].

### 3.8. Leaflets

**Figure 9:**
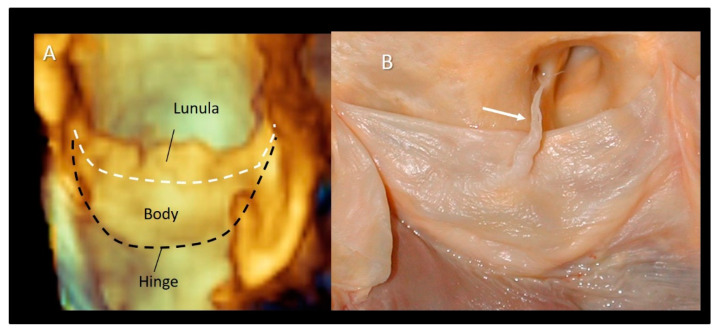
(**A**) The 3D TEE magnified image showing the bird’s nest appearance with the lunula, body, and hinge line. (**B**) Anatomic specimens showing the Lambl’s excrescences (arrow).

The aortic leaflets undoubtedly represent the “*working component*” of the aortic root. The normal aortic valve comprises three leaflets with slight differences in size and shape mirroring the coronary sinuses: usually the non-coronary leaflet (NCL) is the largest and the left coronary leaflet (LCL) the smallest, while the size of the right coronary leaflets (RCL) is in between the other two. Each leaflet has several distinct anatomical regions. The hinge line has a crescent shape being part of the crown-shaped annulus [14,15]. The body is the skeleton of the leaflet bearing the most force in diastole when the valve is closed. The *lunula*, which corresponds to approximately 30% of the leaflet total area, forms the appositional surface between leaflets. In the middle of the lunula at the free margin, there is a thickened nodule called the *Nodule of Arantius*. During diastole, each leaflet, together with the corresponding sinus, takes the appearance of a bird’s nest. The insertion line of the leaflet is approximately 1.5 times longer than the length of the free margin, and the height (base-margin) ranges between 12 and 18 mm. A particular structure is often present on the aged leaflets: the Lambl’s excrescences (LEs) [44,45,46,47]. LEs are fibrous strands typically occurring at coaptation surfaces of the leaflets. These filiform fronds take origin from small thrombi and fibrin deposition on the endocardial surfaces (where the valve margins contact) as a result of micro injuries. Over time, this fibrin–thrombi depot overgrows in thin papillae and eventually in thin, mobile strands made up of a dense core of collagenous and elastic fibrils enclosed by endothelium. LEs are usually found incidentally in otherwise asymptomatic aged patients and only rarely it can be the cause of stroke or coronary artery obstruction/occlusion as opposed to the fibroelastomas [48] (Figure 9).

As for atrio-ventricular valves, aortic leaflets are roughly composed of three layers covered, on both sides, by endothelium. This latter is a natural barrier for inflammatory cell infiltration or lipid accumulation and regulates permeability and cell adhesions by paracrine signals. The so-called *lamina fibrosa* is a layer of dense collagen fibers located beneath the aortic surface of the leaflets and arranged in a circumferential fashion. The collagen fibers of the lamina of each leaflet curve into the wall of the corresponding sinus, merging with elastic and muscular layers. This structural architecture reinforces the concept that the leaflet and sinus may be considered as an anatomical and functional unit. Individual leaflets are therefore suspended from their relative sinus almost totally by means of lamina fibrosa. Interestingly, these collagen fibers have an “undulating” radial arrangement. During the cardiac cycle, the leaflet area is about 50% larger in diastole compared to systole. Given its particular collagen fiber arrangement, the fibrosa can be easily “stretched” in diastole and “wrinkled” in systole. *The ventricularis* is made up predominantly by elastic fibers. This elastic lamina, being stretched in diastole and recoiled in systole, concurs with the fibrosa to expand the surface of the leaflets under diastolic pressure in order to prevent leaks through the coaptation surface. Interesting is the role of the *spongiosa*, a layer of loose connective tissue situated in between the other two layers. The spongiosa is the most complex of the three layers, being made up by sparse elastin and collagen fibers and by a rich extracellular matrix comprising complex glycoproteins, a network of nerve fibers and capillaries, and an array of different cell types, among which the most representative are the valvular interstitial cells (VICs). Each component of the spongiosa confers specific physical properties. *Elastin* allows recoil after each deformation, and *glycoproteins* provide a sort of “lubricant”, which reduces mechanical and frictional stress. Final VICs are essential in the regeneration process and damage repair. The continuous renewal of all components is one of the secrets of the leaflet’s longevity [48].

### 3.9. The Sinutubular Junction

The sinutubular junction (STJ) is the distal part of the aortic root, between the aortic sinuses and the tubular segment of the ascending aorta. Contrary to the other “virtual” rings, the STJ is a true ring made up by circumferentially aligned fibro-elastic lamellae as a mild waist. This fibrous-elastic ring is more pronounced in the proximity of the attachments of the three commissures. Because of the strict proximity of the STJ with the commissures, this fibrous-elastic ring is an integral part of the valvular mechanism. The aortic valve insufficiency associated with the aneurysm of the aortic root, is due to the stretch of the STJ and commissures (which are dragged away from each other), that causes the tethering on the aortic leaflets and, as a consequence, a reduced systolic and diastolic excursion. As mentioned above, the STJ plays a role in generating the vortex behind the aortic leaflets [14,15].

### 3.10. The Coronary Ostia

**Figure 10:**
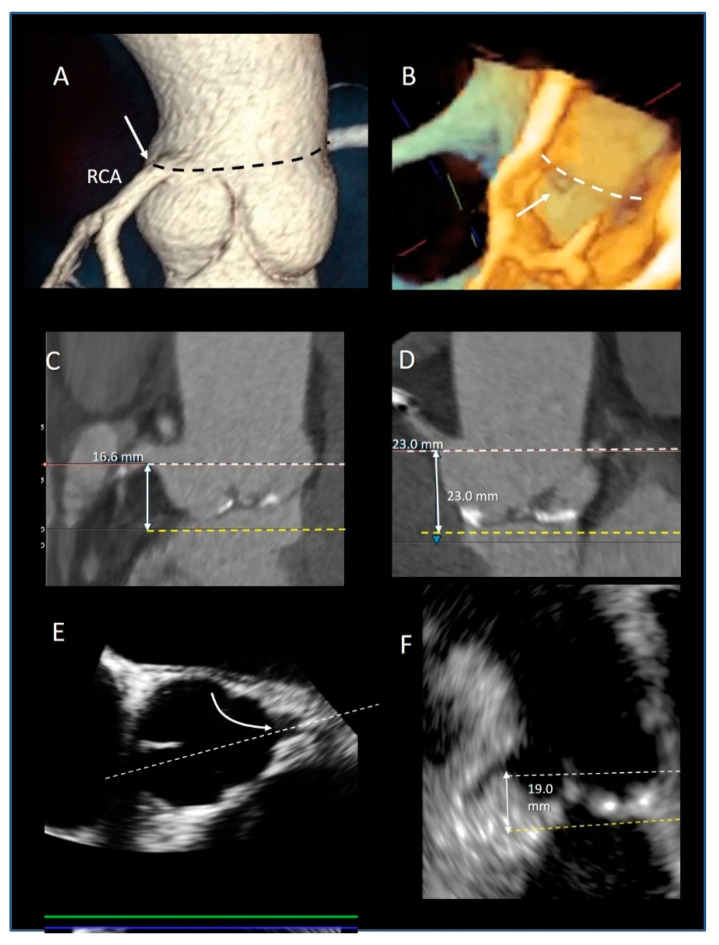
(**A**) CT Volume rendering modality showing the origin of right coronary artery arise at the level of sinutubular junction (black dotted line). (**B**) The 3D TEE longitudinal cross-section showing the orifice of coronary artery (arrow) at level of sinutubular junction (white dotted line). (**C**,**D**) CT multiplanar image modality showing the measurements of the distance (double-headed arrow) between the coronary ostia (white dotted line) and the virtual annulus (yellow dotted line). (**E**) The 2D TEE multiplanar reconstruction derived by 3D data set in short-axis view of the aorta showing the orifice of LAD (curved arrow). The white dotted line indicates the longitudinal cross-section plane that shows the image of the panel. (**F**) From this image, the distance between the coronary ostia (white dotted line) and the virtual annulus (yellow dotted line) can be measured (double-headed arrows).

The preferential location of the coronary ostia is immediately below the STJ, arising from the maximum curvature of the sinus. Muriago et al. studied 23 normal hearts from autopsies in adults. They found that the left coronary artery arose within the left coronary aortic sinus in 16 (69%) specimens, above the sinutubular junction in 5 (22%), and at the level of the junction in 2 (9%). The right coronary artery arose within the right coronary aortic sinus in 18 (78%) specimens, above the junction in 3 (13%), and at the level of the junction in 2 (9%). Interestingly, accessory coronary orifices are found in the majority of the right coronary sinuses [49] (Figure 10A,B).

The position of the coronary ostia above the virtual annulus has relevant clinical implication both in aortic root replacement, where the coronary ostia reimplantation is a key stage of the operation, [50] and in TAVI procedures [51,52], also including the valve in valve implantation [53]. In TAVI procedures, the coronary ostia may be obstructed either by the implanted aortic stent valve itself, or by the displacement of leaflet calcifications into the coronary lumen. The incidence of this complication in patients treated with balloon expandable devices is higher (1.1%) than in those treated with self-expanding valves (0.4%) [54]. The occurrence of coronary obstruction is also high in valve-in-valve TAVI procedures (2.3%), especially when the failed bioprosthesis features externally-mounted valve leaflets [53]. In the absence of defined guidelines, the currently recommended minimum distance of the coronary ostia from the aortic annulus is 10 mm [54]. Although CT is the main modality to identify coronary ostia [55] (Figure 10C,D), 3D TEE is also a valid alternative [56] (Figure 10E,F). Thus, a pre-procedural evaluation with CT or 3D TEE of the coronary ostia position (relative to the virtual annulus and aortic leaflets) is mandatory to minimize the risk of this rare but potentially life-threatening complication.

## 4. Conclusions

The 3D-TEE, CT, and CMR provide excellent images of a “dynamic” aortic root anatomy, both in two- and three-dimensional format that admirably joins structure and function. This review describes the components of the aortic root, specifically the sinutubular junction, the crown-shaped aortic annulus, the Valsalva’s sinuses, the leaflets, the STJ, and the position of the coronary ostia as revealed by non-invasive imaging techniques. Nowadays, new portable echocardiographic machines, as small and light as a cell phone, are available and likely will substitute the stethoscope in the coming years showing the heart and valves in real time (does the word “stethoscope” not derive from the ancient Greek words στηθόυς (stetheos), which means chest, and σκοπεω (skopeo), which means look into?). With the highest spatial resolution power and ultra-low radiation exposure, the new CT machines are able to depict cardiac anatomical details that were unthinkable just a few years ago. Moreover, latest advances in 3D CT reconstruction techniques can now visualize the very nuance of the 3D living anatomy with greater spatial resolution and wide field of view compared with echocardiography [12,13]. Finally, the new CMR offers superb anatomic imaging, tissue structures, and flow imaging in a unique examination. We strongly believe that in the third millennium, medical students and, in particular, cardiologists will learn the cardiac anatomy using these powerful imaging tools. We hope that this review will also serve this aim.

## Figures and Tables

**Table 1:** Terms used to describe the aortic root components.

Name	Meaning	
Ventricular–arterial junction	The term ventricular–arterial junction describes the border between the ventricular myocardium and the fibroelastic structure of the aortic root. Contrary to the right AV junction, where the pulmonary root is entirely supported by the muscular infundibulum, only the left and the right coronary sinuses are partially supported by the myocardium (see text), being the remaining extent of the aortic root supported by fibrous tissue (MS = membranous septum; see text and references [12,13]).	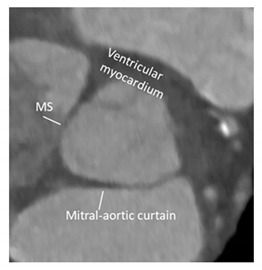
Cusps, leaflets	The term “cusps” refers to the moving parts of the aortic root. When seen in closed position from the ventricular perspective, this component is similar to the surface of a molar tooth (called cusp). The term is used to describe the structure of the valve (i.e., unicuspidal, bicuspid, and tri-cuspid). Literally the term indicates a pointed end where two curves meet. In the aortic root, it indicates an intact interleaflet triangle with its apex reaching the sinutubular junction. The term leaflet means “small leaf”, which describes a thin, pliable layer. This term perfectly fits the leaflet aspect. N = non-coronary, L = left coronary, R = right coronary leaflets/cusps.	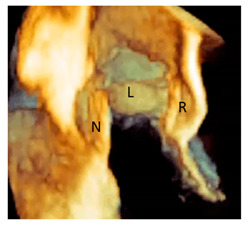
Commissures	In the anatomy of the aortic root this term refers to the most distal area where the insertions of the leaflets on the aortic wall join each other.	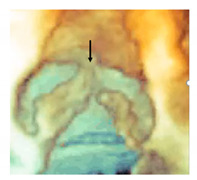
Aortic annulus	The aortic annulus is the three-dimensional line that follows the hinge line of the leaflets on the aortic wall. This line of dense connective tissue has a crown-shaped appearance (white dotted line).	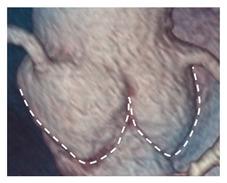
Virtual or “echocardiographic” annulus	This term refers to a circumference that joins the lowest points of the leaflet insertion. Although neither anatomically or histologically recognizable, this term has become relevant in the TAVI era. Measurements of this virtual basal plane are used for the sizing of the valve in tricuspid valves. This virtual annuls does not have an anatomic counterpart.	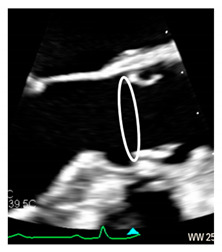
Surgical annulus	Surgeons fix the prosthetic valves on a circular area lying between the nadirs of the sinuses and midway to the commissures (the aortic prostheses have a flat sewing ring). The term *“surgical annulus*” refers to this “ring area” and provides a precise reference point when the prosthesis is sutured on “supra” annular position.	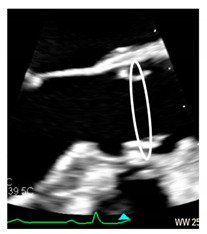

**Table 2:** Functions of the aortic root.

Functions of Aortic Root
Allowing the transit of a significant amount of blood with a minimum gradient between the left ventricle and the aorta
nsuring a wide flow variation (up to 5 times)
Preventing significant back flow, having, at the same time, a robust structural integrity to withstand the aortic pressure
Enabling an optimal coronary perfusion
Preserving leaflet integrity
